# Case Illustrative of Prejudicial Influence of Quinine Improperly Administered

**Published:** 1862-04

**Authors:** Edward Lawrence

**Affiliations:** Hopper’s Mills, Henderson County, Illinois


### CASE ILLUSTRATIVE OF PREJUDICIAL INFLUENCE OF QUININE IMPROPERLY ADMINISTERED.

By DR. EDWARD LAWRENCE,
*Hopper's Mills, Henderson Co., Ills.*

[This paper was received some time since, but was mislaid.]

A little boy aged three years, with a very large brain, was taken with fever on Wednesday night. Thursday morning the fever had somewhat abated, and the mother prevailed on the child to go to the breakfast table, but the boy said he was too sick to eat. While at the table, the child was seized with a convulsion, and fell off the chair on the floor. Reaction took place, and the parents sent for Dr. ——, who came immediately, and said the boy had the ague, and prescribed twelve powders of Sul. Quinine; one to be taken every two hours, commencing about ten minutes after reaction from a convulsion.

I was requested as a friend to step in. I found the child in high fever, skin dry and hot, with a great determination of blood to the brain; pulse quick, hard, tense and incompressible; pupils dilated; tongue coated in the center, with edges and tip red; abdomon enlarged and hard, showing a retention of urine.

In about fifteen minutes after Dr. —— gave a quinine powder the patient had another convulsion. They gave four of the powders, and the patient had four convulsions.

In the afternoon I was called to see the patient. I found all of the symptoms aggravated. The patient was in a comatose state, with great determination to the brain. I ordered cold applications to the head, mustard draft to the spine, used the catheter, and prescribed x gr calomel and 1 gr James' powder. After waiting eight hours, ordered a dose of castor oil. In due time the bowels were moved, but no action on the liver. I repeated the dose, but with the same result. I then prescribed four powders, containing each 1. gr Calomel, ½ gr. Ipec. and ½ gr. Opii; one to be given every two hours; which had a decided action on the liver, resulting in amelioration of all the urgent symptoms. Together with the above treatment, I gave Sweet Spts. Nitre and Syrup Ipec. every two hours, with the view of controlling the action of the heart, equalising the circulation, and giving a determination to the skin.

Other urgent symptoms set in that appeared almost insurmountable. Although the pulse became soft, full and compressible, there was still a tendency to determination to the brain. The determination had been so great that paralysis of the bowels and bladder was the result. I had to resort to catheterism every day. The bowels became tympanitic from loss of nervous influence. I gave large doses of Turpentine every night, bathed the bowels with the same, applied the bandage, and kept cold applications to the abdomen I continued the above treatment, fulfilling every indication as it arose, until the seventh day of the fever, when the remission became distinct. Then I prescribed eight powders, each containing 1½ grs. Sul. Quinine, ½ gr. Ipec., ½ gr. Chlor. Potash; one powder to be given every three hours during remission. Under this prescription the child appeared to rally, although the accumulation of gas in the bowels was very great, I could relieve it by introducing a large sized elastic male catheter, and warm injection of rain water, salt and castile soap.

I continued the Quinine prescription for three days, when the child was restored to health. The healthy function of the bowels and bladder was gradually restored. The tenth day from the commencement of the fever I discharged the patient.

The indications of cure in this case I thought were, first—to relieve urgent symptoms; second—to draw off irritation from the brain, control the action of the heart, and equalise the circulation; third—to establish healthy secretion; fourth—to break up the paroxysm. I fulfilled the first by resorting to catheterism, and cold applications to the head; second, by sinapisms to the spine, and administering a cathartic arterial sedatives and diaphoretics; third, by administering small doses of Calomel, Ipec. and Opii; fourth, by tonics, diaphoretics and saline diuretics.

Did the course I pursued have a tendency to cut the fever short or prolong it?

The previous treatment I thought was wrong from the fact that there was such a great determination to the *brain*. I was under the impression that the Quinine only caused a greater determination to that organ.

